# Involvement of Peripheral Nerves in the Transgenic PLP-α-Syn Model of Multiple System Atrophy: Extending the Phenotype

**DOI:** 10.1371/journal.pone.0136575

**Published:** 2015-10-23

**Authors:** Daniela Kuzdas-Wood, Regina Irschick, Markus Theurl, Philipp Malsch, Norbert Mair, Christine Mantinger, Julia Wanschitz, Lars Klimaschewski, Werner Poewe, Nadia Stefanova, Gregor K. Wenning

**Affiliations:** 1 Department of Neurology, Medical University of Innsbruck, Innsbruck, Tirol, Austria; 2 Department of Anatomy, Histology and Embryology, Division of Clinical and Functional Anatomy, Medical University of Innsbruck, 6020 Innsbruck, Austria; 3 Department of Anatomy, Histology and Embryology, Division of Neuroanatomy, Medical University of Innsbruck, 6020 Innsbruck, Austria; 4 Department of Cardiology and Angiology, Medical University of Innsbruck, Innsbruck, Tirol, Austria; 5 Department of Physiology and Medical Physics, Medical University of Innsbruck, Innsbruck, Tirol, Austria; University of Pittsburgh School of Medicine, UNITED STATES

## Abstract

Multiple system atrophy (MSA) is a fatal, rapidly progressive neurodegenerative disease with (oligodendro-)glial cytoplasmic α-synuclein (α-syn) inclusions (GCIs). Peripheral neuropathies have been reported in up to 40% of MSA patients, the cause remaining unclear. In a transgenic MSA mouse model featuring GCI-like inclusion pathology based on PLP-promoter driven overexpression of human α-syn in oligodendroglia motor and non-motor deficits are associated with MSA-like neurodegeneration. Since α-syn is also expressed in Schwann cells we aimed to investigate whether peripheral nerves are anatomically and functionally affected in the PLP-α-syn MSA mouse model.

**Results:**

To this end, heat/cold as well as mechanical sensitivity tests were performed. Furthermore, in vivo and ex vivo nerve conduction and the G-ratios of the sciatic nerve were analyzed, and thermosensitive ion channel mRNA expression in dorsal root ganglia (DRG) was assessed. The presence of human α-syn in Schwann cells was associated with subtle behavioral impairments. The G-ratio of the sciatic nerve, the conduction velocity of myelinated and unmyelinated primary afferents and the expression of thermosensitive ion channels in the sensory neurons, however, were similar to wildtype mice.

**Conclusion:**

Our results suggest that the PNS appears to be affected by Schwann cell α-syn deposits in the PLP-α-syn MSA mouse model. However, there was no consistent evidence for functional PNS perturbations resulting from such α-syn aggregates suggesting a more central cause of the observed behavioral abnormalities. Nonetheless, our results do not exclude a causal role of α-syn in the pathogenesis of MSA associated peripheral neuropathy.

## Introduction

Multiple system atrophy is a fatal, sporadic, adult-onset, neurodegenerative disease, characterized by parkinsonism, cerebellar ataxia, and autonomic failure (AF) in different combinations [1, 2]. The mean age of disease onset is around 56 years and survival after disease onset lies between 6 and 9 years [3]. The prevalence is 1.9–4.9/100,000 and the incidence is 3/100,000/year in the population over 50 years, thus MSA meets orphan-disease status (Orpha number: ORPHA102)[4]. Depending on the predominant motor presentation, MSA can be classified into a Parkinson variant (MSA-P) associated with striatonigral degeneration (SND) and a cerebellar variant (MSA-C) defined by olivopontocerebellar atrophy (OPCA)[5].

Together with Parkinson’s Disease (PD) and dementia with Lewy bodies (DLB), MSA belongs to the spectrum of α-synucleinopathies. In PD and DLB, α-syn-positive aggregates are mainly found in neurons, whereas the predominant location for α-syn accumulation in MSA is the cytoplasm of oligodendrocytes forming glial cytoplasmic inclusions (GCIs) [6].

MSA research has mostly focused on the central nervous system (CNS), and only recent studies address pathological alterations of the peripheral nervous system (PNS) [7–14]. Up to 40% of MSA patients develop a peripheral neuropathy (PNP) [7–11]. The pathogenesis of MSA is still unclear, however, peripheral α-syn pathology may play a role [11]. One study reported α-syn-immunoreactivity in neuronal cytoplasm and processes of sympathetic ganglia in 42,3% of MSA patients [12]. MSA patients with α-syn-immunoreactive structures in sympathetic ganglia or neuronal cytoplasm had significantly longer disease duration at the time of the examination than patients lacking α-syn pathologies. None of the tissue samples from healthy individuals and only 4% of patients with amyotrophic lateral sclerosis cases show comparable PNS α-syn pathology [12]. Until now, reports about the presence of α-syn in Schwann cells were controversial. Even though α-syn was reported to be expressed in Schwann cells of healthy controls [13], no α-syn-immunoreactive structures could be found in the Schwann cells of MSA patients in another study [12]. However, a recent study investigated cranial and spinal nerves, peripheral ganglia and visceral autonomic nervous system and found α-syn inclusions in the cytoplasm of Schwann cells in 12 out of 14 MSA patients, but not in any of the healthy controls [14] thereby supporting the findings of the current study.

Transgenic (tg) PLP-α-syn MSA mice with specific oligodendroglial overexpression of human α-syn (hα-syn) under the control of the proteolipid protein (PLP) promoter have proven useful to study MSA-related pathogenic mechanisms [15]. The tg PLP-α-syn MSA mouse model features GCIs as well as mild, progressive striatonigral degeneration, microgliosis, motor impairment and signs characteristic for autonomic failure [15–21]. The current study focused on the role of hα-syn in the PNS of the PLP-α-syn mouse model. Increased sensitivity to heat and cold stimuli could be detected in the PLP-α-syn mouse model of MSA compared to age-matched healthy controls. The fact that nerve conduction velocity, myelin thickness and mRNA expression levels of the thermosensitive ion channels *Trpa1* and *Trpm8* in the DRG did not differ between the two groups suggests a different cause of this hypersensitivity in the PLP-α-syn mouse model.

## Materials and Methods

### Animal groups

All behavioral tests and experimental procedures were performed in accordance with ethical guidelines and Austrian law (Federal Ministry of Science, Research and Economy), and comply with international laws and policies (Directive 2010/63/EU of the European parliament and of the council of 22 September 2010 on the protection of animals used for scientific purposes; Committee for the Update of the Guide for the Care and Use of Laboratory Animals, Council NR, 2010). All efforts were made to minimize animal suffering, to reduce the number of animals used, and to utilize alternatives to in vivo techniques. The same group of animals was used for behavior and nerve conduction velocity measurements to reduce the total number. To reduce stress and pain for the animals, only one of the conductance measurements was performed at the 12/15months time-points. During the experiments all efforts were made to reduce stress and pain for the animals. Animals' well being was monitored by well-trained animal care-takers. Animals were housed under 12-hour light/dark-cycle with free access to food and water when not in experimental sessions which required otherwise.

**Fig 1:**
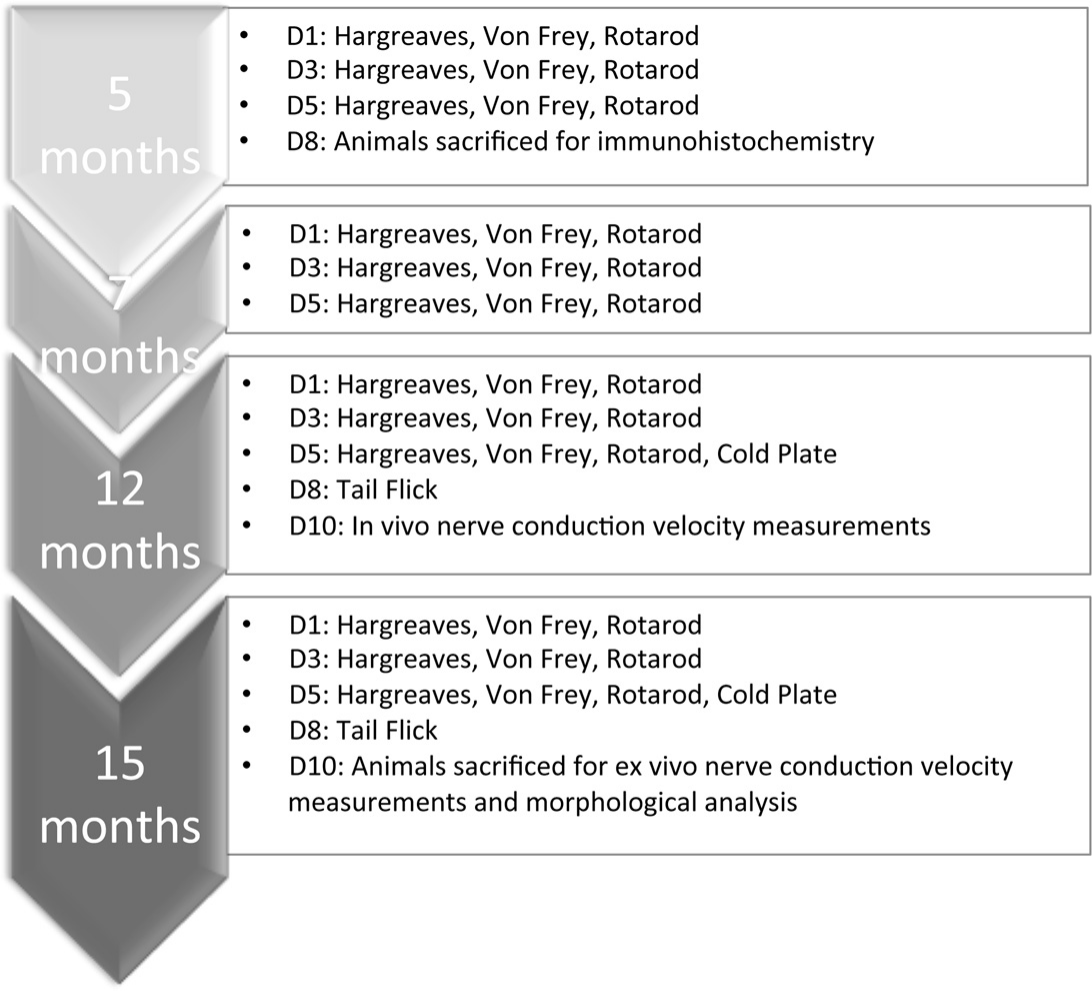
Experimental design. Fig 1 illustrates experimental design and gives an overview of the behavioral tests performed at each time-point (5–15 months) and which days (D1—D10).

Tg male PLP-α-syn mice expressing hα-syn under the control of the oligodendroglial proteolipid protein (PLP) promoter in C57BL/6-background (previously described [15, 20, 22]) and age-, sex- and background-matched wildtype (wt) healthy control group were individually housed starting 48 h before the onset of the behavioral tests. Behavioral tests were performed at 5, 7, 12 and 15 months of age. Cold plate and Von Frey tests were performed once per day, and for Hargreaves, 5 values were obtained for each hind paw and the mean calculated. The same set of animals was used for behavior and nerve conduction velocity measurements to reduce the total number. To reduce stress and pain for the animals, only one of the conductance measurements was performed at the 12/15months time-points. The total number of animals used for this study was 21 of each genotype (8 for the 5 months behavior and sciatic nerve immunohistochemistry, 8 for the rest of the functional read-outs and DRG immunohistochemistry, 5 for qPCR). See Fig 1 for an overview of the study design.

### Behavioral tests

#### Evaluating mechanical sensitivity—Von Frey test

In order to address mechanical sensitivity mice were allowed to habituate in the test plastic chamber with a wire mesh floor for 1 h before start of the test period. Mechanical sensitivity at the hind-paw was determined by probing the plantar side of the hind paw with calibrated von Frey monofilaments (Bioseb, In vivo research instruments) with bending forces between 2.8 and 45.3 mN. The withdrawal threshold was determined by increasing and decreasing stimulus intensity on the basis of the up-down method, starting with 11.4 mN [23–25]. The assessment was done at 5, 7, 12 and 15 months of age with a tg PLP-α-syn MSA group and age-, sex- and a background-matched wildtype (wt) healthy control group by the same blinded examiner.

#### Heat-sensitivity-measurement—Hargreaves test and tail flick test

The Hargreaves test was used to assess sensitivity to thermal stimulation as described previously [26, 27]. Briefly, a radiant heat source delivering a heat stimulus was focused under the plantar surface of the hind-paw. The time from initiation of the stimulus until paw withdrawal was measured by an algesiometer (Ugo Basile, Varese, Italy). Each hind-paw was tested at least three times per trial with at least 60 seconds between trials. Over a period of five days, three trials were performed with each mouse being tested every other day. The mean withdrawal latency was calculated [27].

Besides the Hargreaves test, heat sensitivity was also assessed using the tail flick test [28] at 12 and 15 months of age. The temperature at which the animals started rapidly flicking their tail was taken as the read-out for this test.

#### Cold plate test

For the cold plate test, mice were adapted to 25°C for five minutes before starting the three minute test period on the 0°C cold plate (both Ugo Basile, Varese, Italy) [29]. The latency until the first jump as well as the total number of jumps was recorded.

#### Rotarod

In order to assess balance and coordination abilities [30, 31] mice were transferred to an accelerating rotarod with acceleration from 4 to 40 rpm with a cut-off time of ten minutes (Acceler Rota-Rod 7650, Ugo Basile, Varese, Italy) and the time spent on the rod was recorded. At each time-point, animals were tested on three different days. The mean time spent on the rotating rod was used for analysis [30, 31]. A 600 s cutoff time was used. No difference in the learning curve was observed over the three-day trial period between the groups.

### *In Vivo* Nerve Conduction Measurement

Recordings of sciatic nerve motor and sensory waves were performed in sedated 12 month-old wt and tg mice. A constant body temperature of 37°C was maintained automatically using a heating pad equipped with a rectal thermo probe. Sciatic nerves were stimulated at two sites using paired percutaneous monopolar needle electrodes; the electrodes were inserted at the sciatic notch and distal at the level of the ankle. A surface electrode was placed at the base of the tail to serve as a ground electrode. Supramaximal square wave stimulations of fixed duration (0.1 millisecond) (Teca TD-10; Oxford Instruments, Oxford, UK) were applied to the nerve, and the resulting muscle compound action potentials (CMAPs) were recorded in the interosseous muscles of the ipsilateral foot with two needle electrodes. The mean latency difference between three pairs of the distal and proximal CMAP responses was determined and combined for each data point. Motor nerve conduction velocity (MCV; meters per second) was calculated by dividing the interelectrode distance between the two stimulation sites (in millimeters) as measured with calipers by the mean latency difference (in milliseconds) of the CMAPs.

### Ex vivo nerve conduction velocity measurement

An in vitro skin nerve preparation was used to investigate the properties of the afferent nerve fibers innervating the skin of the mouse dorsal hind-paw as previously published [32, 33]. The preparation was superfused (15 ml/min) with an oxygen-saturated modified synthetic interstitial fluid solution containing (in mM) 108 NaCl, 3.48 KCl, 3.5 MgSO4, 26 NaHCO3, 1.7 NaH2PO4, 2.0 CaCl2, 9.6 sodium gluconate, 5.5 glucose, 7.6 sucrose at a temperature of 31 ± 1°C, and pH 7.4 ± 0.05. Action potentials of single sensory neurons were recorded extracellulary from fine filaments dissected from the saphenous nerve, amplified (5000-fold), filtered (low pass 1 KHz, high pass 100 Hz), visualized on an oscilloscope, and stored on a PC-type computer with Spike/Spidi software package [34]. The stimulation electrode was positioned close to the transition to the skin. The amplitude [V] of the stimulus (square pulse 1 ms) was increased until every stimulus triggered an action potential. From the interelectrode distance and the delay time the conduction velocity [m/s] was calculated. Fibers were characterized as unmyelinated (C) according to their conduction velocity (< 1.0 m/s) [31–33]. Two recordings were performed on each of the three mice in the ex vivo test on explants of the left and right hind paw.

### Immunohistochemistry, immunofluorescence and image analysis

After the behavioral analyses were completed, animals were sacrificed by cervical dislocation. The sciatic nerve (s.n.) of five month-old tg and wt animals were isolated and immersion fixed with 4% paraformaldehyde (PFA, Sigma-Aldrich, St.Louis, USA) overnight at 4°C. Afterwards they were put in 30% sucrose for one night before they were frozen with 2-Methylbutane on dry ice. Nerve samples were sliced into 10 μm thick sections with a freezing microtome (Leica, Nussloch, Germany) and mounted on slides immediately during tissue slicing and stained on slides. Blocking solution (0.01M PBS + 5% serum (species that secondary antibody was generated in) + 1% bovine serum albumin was applied for one hour before incubation with the antibody solution. The following antibodies were used in this study: monoclonal rat-anti-human-α-syn (15G7, Enzo Life Science, Farmingdale, NY, USA), monoclonal mouse-anti-CNPase antibody (Abcam, Cambridge, UK), polyclonal rabbit-anti-S-100 (Sigma-Aldrich, St.Louis, USA), rabbit-anti-β-III-tubulin antibody (Abcam, Cambridge, UK) in combination with the corresponding secondary antibodies: goat anti-mouse IgG Alexa 546, goat-anti-rabbit IgG Alexa 546, goat-anti-rat IgG Alexa 488 (Invitrogen, Eugene, Oregon, USA). The dorsal root ganglia (DRG) of tg PLP-α-syn and wt mice were isolated at 15 months of age and processed like the s.n. samples for immunofluorescence labeling. Briefly, they were stored in 4% ice-cold PFA overnight, then transferred to 30% sucrose solution for one night before freezing in 2-Methylbutane and storage at -80°C until slicing. DRGs were sliced on the same freezing microtome into 12 μm thick slices and collected on slides. Immunofluorescence labeling was performed with antibodies for β-III-tubulin to label neuronal cells and 15G7 to identify hα-syn. Confocal images were obtained with a Leica TCS SP5 laser scanning microscope using a 63x oil objective (N.A. 1.4) (Leica Microsystems, Wetzlar, Germany).

To ensure the specificity of the immunolabeling, only well-established antibodies were used. Wildtype samples were included to confirm the specificity of the antibody against human α-syn. Furthermore, all stainings were performed without primary antibody to exclude unspecific binding of the secondary antibodies.

### Morphological analysis of the myelin thickness

For morphological analysis, the s.n. of 3 tg and 3 wt animals of 15 months age were isolated post mortem and immersion fixed with 4%PFA + 30% picric acid and nerves were stored in PFA-picric-acid solution (Sigma-Aldrich, St.Louis, USA) with 2.5% glutaraldehyde overnight and then transferred to 0.1 M PB. For ultra-thin sections, nerves were embedded in epoxy resin. Nerves were sliced on an ultra-microtome into 70 nm thick ultra-thin slices and stained with toluidine blue solution (0.1 g Toluidine blue in 100 ml 2.5% sodium carbonate solution). For quantification of the G-ratio, microphotographs were taken with 100x magnification. The inner and outer axon diameters were measured using imageJ software (NIH,-Bethesda, Maryland, USA) the calculation of the G-ratio was performed in Microsoft Excel. Axons were divided into three categories according to previous reports [35]: 1) thin: <5 μm, 2) medium: 5 μm—8 μm and 3) large: 8 μm -12 μm and the ratio of inner/outer axon diameter was calculated for calculated from nerve samples of 15 month-old animals. 5 microphotographs were taken from each animal to obtain approximately 300 G-ratio values per animal.

### Quantitative RT-PCR

For the qPCR experiment, five 12 month-old animals were used per group (wt and tg). For analysis of mRNA expression levels, DRG explants were harvested from lumbar L1 to L6 spinal segments. DRG explants were lysed with a Tissue Lyser II (Quiagen, Netherlands) and total RNA was isolated by using peqGOLD TriFast (PeqLab, VWR International GmbH, Erlangen, Germany) as previously published [36, 37]. Reverse transcription to cDNA was performed using MuLv Reverse transcriptase (2.5 U/μl, Applied Biosystems, MA, USA) with Random Hexamer primers (10 ng/μl), RiboLock (2 U/μl), 1x Taq Buffer (all Thermo Scientific, MA, USA), MgCl2 (5 mM) and dNTPs (1 mM, both Fermentas, MA, USA). cDNA samples were analyzed for expression of target genes by quantitative real-time Taqman PCR using Taqman 5´ nuclease assays (all Applied Biosystems, MA, USA). The following exon spanning assays were used: Mm01352363_m1 (Succinate Dehydrogenase Subunit A, *Sdha*), Mm00625268_m1 (transient receptor potential cation channel, subfamily A, member 1, *Trpa1*), Mm01299593_m1 (transient receptor potential cation channel, subfamily M, member 8, *Trpm8*). Reactions were performed in a MicroAmp Fast Optical 96-Well Reaction Plate (Applied Biosystems, MA, USA) using the 7500 Fast Real-Time PCR System (Applied Biosystems, MA, USA) for thermal cycling and real-time fluorescence measurements. The PCR cycle protocol consisted of 10 min at 95°C, and 50 two-step cycles of 15 s each at 95°C and of 1 min at 60°. Each sample was run in triplicates. Threshold cycle (CT) values were recorded as a measure of initial template concentration. Relative expression levels of the target genes were measured as 2-ΔCT with ΔCT = CT(target)–CT(*sdha*)) [38]. Expression of the target gene relative to wildtype samples as calibrator was calculated by 2-ΔΔCT.

### Statistical analysis

Statistical analysis was performed using GraphPad Prism 5.3 Software. Two-way ANOVA was performed for comparisons between tg PLP-α-syn and wt mice of different age groups. Data blotted in graphs are means ± S.E.M. Post-hoc Bonferroni analysis was applied where appropriate. T-test was performed for analysis of nerve conduction velocity and qPCR analysis to compare the two groups. * = p<0.05; ** = p<0.01; *** = p<0.001.

## Results

### Sensorimotor performance is intact in tg PLP-α-syn MSA mice

**Fig 2:**
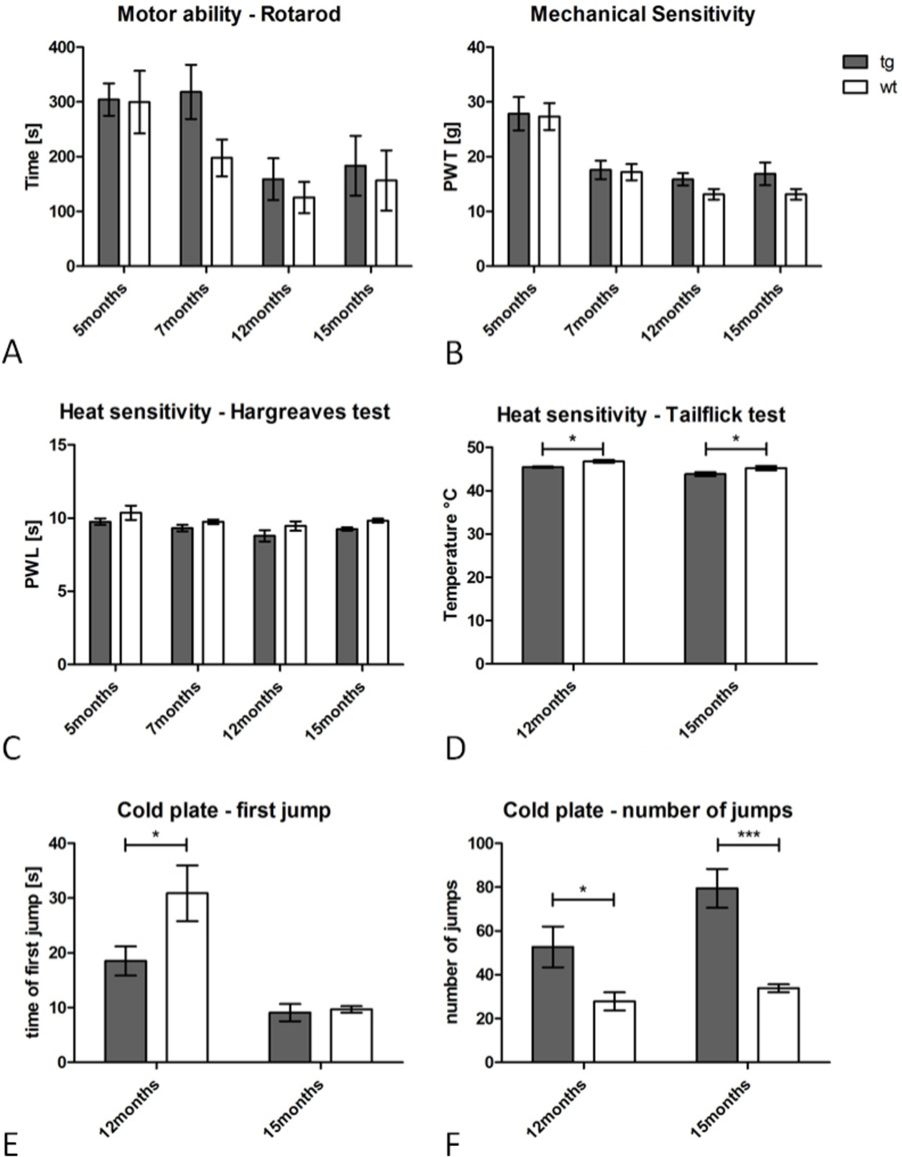
Behavioral characterization of tg PLP-α-syn mice and age-, gender- and background-matched wt controls at different time-points. Investigation of the motor abilities (A) measured as the time spent on the rotarod and mechanical sensitivity (B) indicated by the paw-withdrawal-threshold (PWT) did not reveal any differences between the groups at any time-point. The Hargreaves test for heat sensitivity did not show any significant difference in the paw-withdrawal-latency (PWL) between tg PLP-α-syn and wt animals, however, all values of the tg group were lower than the wt group indicating a trend towards increased heat-sensitivity (C). Analysis of the tailflick test revealed increased heat sensitivity in tg PLP-α-syn animals at 12 and 15 months of age (D) supporting the trend seen in the Hargreaves test. The cold plate-test revealed increased sensitivity to cold stimuli in tg PLP-α-syn animals at 12 and 15 months as seen by the decreased delay of the first jump (E) at 12 months and the increased number of jumps (F) at 12 and 15 months. n = 8, data represent mean values ± S.E.M.

Analysis of motor abilities was assessed using the Rotarod test. The performance of tg PLP-α-syn animals did not differ from the wt group at any time-point indicating intact motor abilities (p >0.05; F_(1, 35)_ = 1.638, n = 8) (Fig 2A). A similar age-dependent decline in the motor performance could be observed in both groups over time. Also the mechanical sensitivity measured with Von Frey bristles did not reveal differences between the groups throughout the ten-month investigation period (p >0.05; F_(1, 35)_ = 2.055, n = 8, Fig 2B).

### Increased heat- and cold-sensitivity in tg PLP-α-syn MSA mice

The Hargreaves test for heat sensitivity did not yield significant differences between tg and wt mice at an age of 5, 7, 12 and 15 months (p >0.05; F_(1, 35)_ = 6.469, n = 8) in the paw-withdrawal-latency (PWL), however, all values of the tg group were lower than the wt group indicating a trend towards increased heat-sensitivity (Fig 2C). Analysis of the tail-flick test (performed at 12 and 15 months) revealed increased heat-sensitivity in the tg PLP-α-syn group at both time-points (p <0.05; F_(1, 35)_ = 11.71, n = 8) (Fig 2D) supporting the trend observed in the Hargreaves test. Investigation of cold-sensitivity at 12 and 15 months in the cold-plate test showed increased sensitivity to cold-stimuli of tg animals as seen in the parameters ‘first jump’ (Fig 2E) as well as ‘number of jumps’ (Fig 2F) (First jump: 12 months: p <0.05, 15 months not significantly different; F_(1, 11)_ = 4.964; number of jumps: 12 months p <0.05, 15 months p<0.001; F_(1, 11)_ = 18.44).

### Intact nerve conduction velocity in tg PLP-α-syn MSA mice

**Fig 3:**
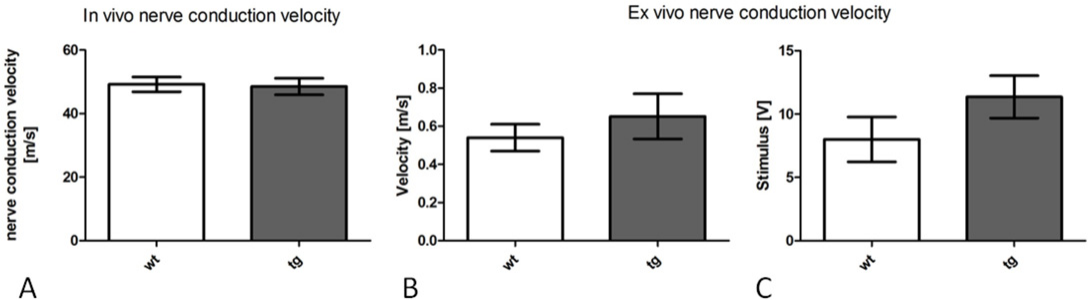
*In vivo* and *ex vivo* measurement of nerve conduction velocity. *In vivo* sciatic nerve conduction velocity was measured in 12 month-old tg PLP-α-syn and wt animals (A). No difference was detected in the conduction properties between the groups. n = 8. Sciatic nerve conduction velocity was measured *ex vivo* in 15 month-old tg PLP-α-syn and wt animals. No difference was detected in the conduction velocity (B) or stimulus strength required (C) between the groups. n = 3, data blotted represent mean values ± S.E.M.

To investigate, whether the increased sensitivity to heat- and cold-stimuli could be caused by alterations in electrical properties of peripheral nerve fibers, the nerve conduction velocity (NCV) was measured at 12 months in vivo and at 15 months ex vivo. Neither of these experiments revealed significant differences between the genotypes in the speed of signal transduction or the voltage required to create stimulation (In vivo NCV [m/s]: p = 0.8465, n = 8, Ex vivo NCV [m/s]: P = 0.0697, Voltage required to create stimulation [V]: p = 0.1960, n = 3) (Fig 3).

### Human α-syn is present in the Schwann cells of tg PLP-α-syn MSA animals

**Fig 4:**
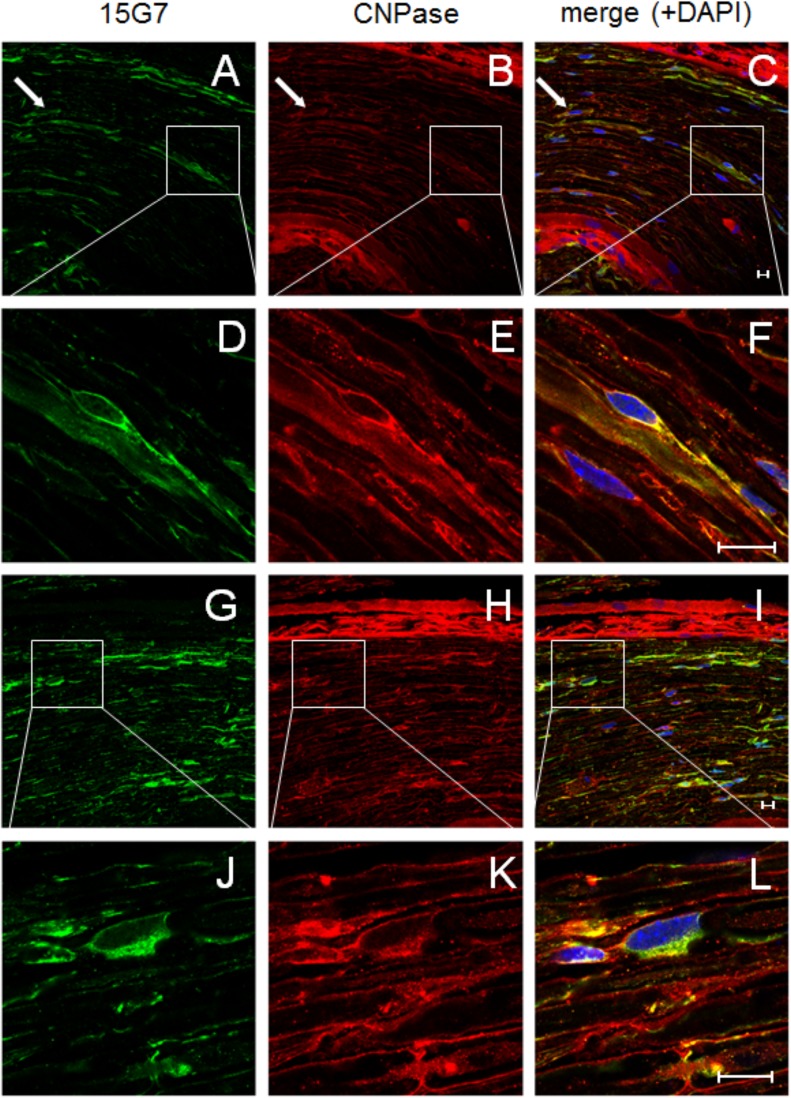
Immunofluorescence stainings of tg sciatic nerve sections stained with the hα-syn marker 15G7 and the Schwann cell marker CNPase. The arrows highlight the co-localization pattern of h—syn (first column, images A, D, G, J) and the Schwann-Cell marker CNPase (second column, images B, E, H, K) in the tissue of tg PLP-α-syn animals, the third column shows the co-labeling of h—syn and CNPase and includes a staining of the nuclei with DAPI (images C, F, I, L). Images D, E, F show magnified areas of A, B, C and J, K, L show magnified sections of G, H, I. scale bar = 10 **μ**m.

**Fig 5:**
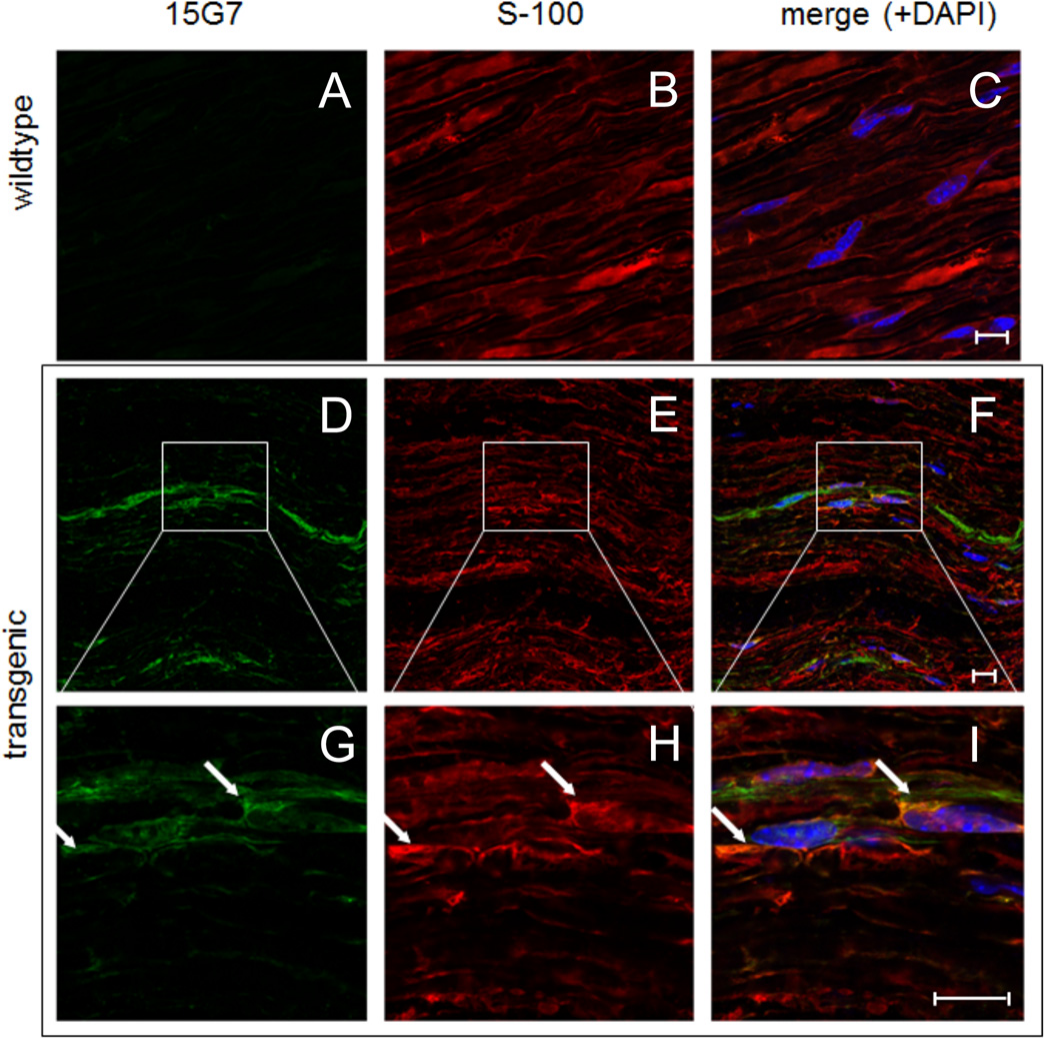
Immunofluorescence stainings of tg sciatic nerve sections stained with the hα-syn marker 15G7 and the Schwann cell marker S-100. The arrows highlight the co-localization pattern of h—syn (first column, images A, D, G) and the Schwann-Cell marker S-100 (second column, images B, E, H) in the tissue of tg PLP-α-syn animals (last 2 rows, images D-I) and wt animals as negative control for the h—syn antibody (first row, images A-C), the third column shows the co-labeling of h—syn and S-100 and includes a staining of the nuclei with DAPI (images C, F, I). Images G, H, I show magnified areas of D, E, F. scale bar = 10 **μ**m.

**Fig 6:**
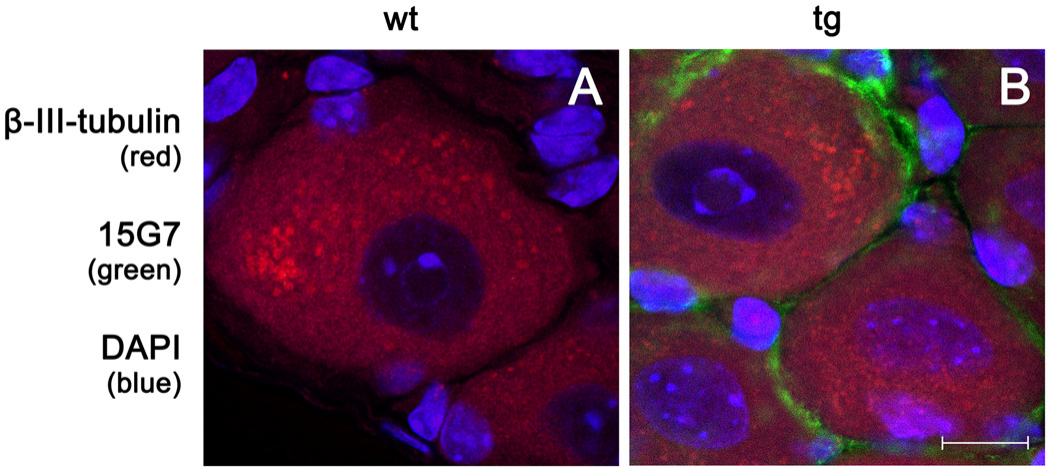
Immunofluorescence labeling of wt and tg DRG sections with the neuronal marker β-III-tubulin and the hα-syn marker 15G7. DRG tissue of wt and tg PLP-α-syn animals stained with the neuronal marker β-III-tubulin (red), the marker for hα-syn 15G7 (green) and DAPI (blue) to label the nuclei. This image confirms the presence of hα-syn in the DRG of the PLP-α-syn mouse model. It can be seen, that the green hα-syn is located around the neurons in red and the markers do not co-localize; scale bar = 10 **μ**m.

**Fig 7:**
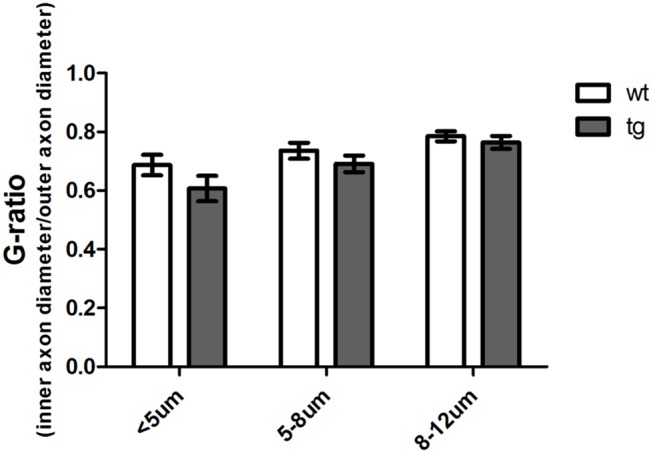
G-ratio of 15 month-old wt and tg PLP-α-syn sciatic nerve sections. The investigation of myelin thickness by calculating the G-ratio (inner axon diameter/outer axon diameter) did not detect differences between tg PLP-α-syn and wt animals in axons thinner than 5 **μ**m, axons between 5 **μ**m and 8 **μ**m and axons between 8 **μ**m and 12 **μ**m (p = 0.1095). Ultra-thin sections of wt and tg PLP-α-syn sciatic nerve tissue have been labeled with toluidine blue, the measurements were performed in microphotographs with 100x magnification. n = 3 animals, 300 fibers of equal distribution analyzed per animal, data represent mean values ± S.E.M.

Analysis of double-labelings with the Schwann cell markers CNPase and S-100 (Figs 4 and 5) in combination with 15G7, a marker for hα-syn, showed clear co-localization of hα-syn in the Schwann cells of five month-old tg PLP-α-syn animals but not in wt mice. Immunolabelling of DRG tissue from tg PLP-α-syn and wt animals with the neuronal marker β-III-tubulin and the marker for hα-syn also confirmed the presence of hα-syn in DRGs (Fig 6). Analysis of the G-ratio (inner axon diameter divided by outer diameter) of tg PLP-α-syn and wt animals revealed no differences between axons of wt and tg animals (Fig 7).

### No difference in expression levels of *Trpa1* and *Trpm8* between wt and tg PLP-α-syn MSA mice

**Fig 8:**
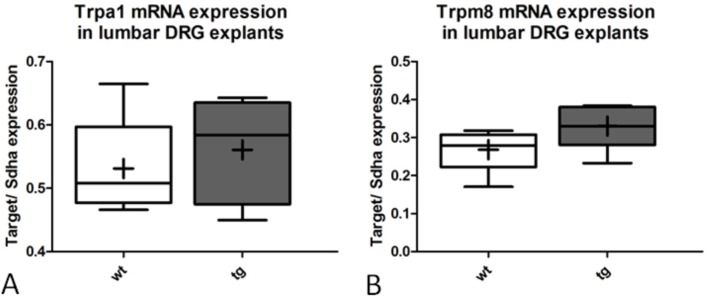
No difference in mRNA expression levels of *Trpa1* and *Trpm8* between wt and tg MSA mice. *Trpa1* and *Trpm8* mRNA levels were similar for wt and tg PLP-α-syn DRG explants. n = 5, Boxes represent median as well as lower and upper quartile; whiskers represent the 10th and 90th percentiles, the mean value is indicated with the ‘+’ symbol.

Cold stimuli can be detected by thermosensitive ionotropic receptors in the peripheral terminals of afferent Aδ- and C-fibers. The ion channels Transient Receptor Potential M8 (TRPM8) and A1 (TRPA1) have been discussed as cold transducer molecules [39, 40]. While TRPM8 primarily transduces innocuous cold stimuli, TRPA1 is activated by noxious cold and has been shown to specifically contribute to cold hypersensitivity under pathological conditions [40–45]. Quantitative Taqman RT-qPCR did not reveal any differences in the expression levels of the two ion channels in lumbar DRG of 12-month old transgenic PLP-α-syn mice or sex-, age- and background-matched wt healthy control group (*Trpa1*: p = 0,5789, *Trpm8*: p = 0,1319 n = 5)(Fig 8).

## Discussion

Neurodegeneration is mainly central in MSA [6], however, increasing evidence suggests a mild but robust involvement of PNS [14]. For example, neurophysiological evidence of a peripheral neuropathy (PNP) is present in 40% of MSA patients [7–9, 46]. The present study investigated whether PNS pathology associated with α-syn deposits occurred in the PLP-α-syn tg MSA mouse model that is characterized by α-syn positive GCIs and MSA-like central neurodegeneration [1, 17, 20, 21, 47, 48].

Animals were observed over an extended period to analyze whether aging contributes to sensory deficits. Motor abilities evaluated with the rotarod test did not show any major disabilities in the tg PLP-α-syn MSA mice at any age indicating a normal function of the motoric components of the sensorimotor system. A decline in motor performance could be observed in both groups to a similar magnitude between five and 15 months of age. More sensitive behavioral tests would have been required to detect subtle gait disturbances as described earlier [20]. The functional characterization revealed intact mechanical sensitivity but hypersensitivity to cold and heat stimuli in the PLP-α-syn mice. The fact that nerve conduction velocity, myelin thickness and mRNA expression levels of the thermosensitive ion channels Trpa1 and Trpm8 in the DRGs did not differ between the two groups might indicate a more central cause of this hypersensitivity in the PLP-α-syn mouse model. However, the excitability of the TRPA1 and TRPM8 channels was not investigated. Therefore, no definite conclusion can be drawn from these results. It is also possible, that other ion channels involved in cold transduction or transformation such as the Nav1.8 or TREK family are responsible for the observed behavioral differences [49–51]. The fact that a difference in the cold plate could be observed, but not in the Hargreaves test, rather suggests differential expression of cold or heat activated ion channels.

Except for the cold-hand sign, which has been described as a red flag in MSA [52, 53] and reports of sensory pain described as burning or coldness sensation [46], there are no further reports indicating a disturbance of heat- or cold-sensitivity in human MSA. Tison and co-workers reported a similar distribution pattern of pain in MSA patients compared to PD patients [46]. In this study, pain was present in 47% of MSA patients, 28% of whom had sensory pain including burning sensations, coldness, tingling or numbness [46]. A later study reported moderate or severe pain in 66% of MSA patients (76% of MSA-P patients, 50% of patients with MSA-C) [54].

The nerve fibers relevant for pain signaling are the slowly conducting C-fibers that are normally unmyelinated and 0.3–1 μm in diameter. However, C-fibers have also been shown to be involved in cold-signaling in mice [55]. Whether C-fibers are specifically affected in MSA remains uncertain. Clinically and neurophysiologically the prevailing finding is that of a sensorimotor axonal neuropathy associated with partial skeletal muscle denervation in up to 40% of patients [9]. Further, the density of sensory afferent and postganglionic sympathetic fibers is mildly reduced [7, 56] and loss of myelinated sural nerve fibers may result in abnormal sensory nerve conduction velocities [7, 10, 57, 58]. Another EMG study reports that neurogenic EMG abnormalities in muscles are more frequently observed in MSA patients than peripheral nerve lesions and suggest involvement of anterior horn cells [59]. The current study is the first to address these parameters in the MSA mouse models but no significant differences to wt control mice were detected.

Since the PLP-promoter drives oligodendroglial overexpression of hα-syn we hypothesized that hα-syn is also expressed in Schwann cells triggering or aggravating peripheral nerve dysfunction. Consistent with our assumption we here demonstrate the presence of hα-syn in Schwann cells of the sciatic nerve and DRG of PLP-α-syn mice. Until recently, reports from human MSA patients on the presence of α-syn in Schwann cells were contradictory, some showing α-syn in healthy individuals, and others detecting α-syn mainly in neuronal cytoplasm and processes of sympathetic ganglia, but not in Schwann cells of MSA patients [12, 13, 60]. However, a recent study confirmed the presence of hα-syn in the Schwann cells of MSA patients and describes so-called Schwann cell cytoplasmic inclusions (SSCIs)[14] thereby supporting the findings of the current study. The authors claim, that both central and peripheral mechanisms contribute to neurodegeneration in MSA, however, further studies will be required to determine the exact role of peripheral hα-syn pathology in the pathogenesis of MSA-associated PNP and pain or sensory disorders—certainly more common factors promoting a PNP such as diabetes, liver and renal failure, excessive alcohol consumption or hematological disorders and drug and toxin exposures need to be excluded [61, 62].

For Parkinson’s Disease, several mechanisms of axonal degeneration have been investigated [63] and several studies in PD models have investigated the role of α-syn in axonal re- and degeneration [35, 64]. The relative preservation of PNS function in tg MSA mice suggests that the behavioral deficits observed in our study may be more attributable to central pain processing pathways affected by the α-synucleinopathy lesions. For example, abnormal striatal dopaminergic transmission and disruption of ascending and descending pathways may play a role [3, 5, 46].

## Conclusion

Here we showed for the first time that PLP-α-syn MSA mice expressed hα-syn not only in oligodendroglia but also in the sciatic nerve and DRG Schwann cells. At the same time, these mice showed mild heat and cold hypersensitivity as a potential correlate of MSA associated pain. Functional, electrophysiological and anatomical data of the present study revealed no other consistent differences to wt mice that explain the temperature hypersensitivity. Our data extend findings on the central neurodegenerative disease process that is known to result from oligodendroglial hα-syn expression in PLP-α-syn MSA mice and support the utility of the PLP-α-syn model as translational testbed for studies on pathogenesis and therapeutic interventions in MSA.
